# Nomenclature of cell-cultivated meat & seafood products

**DOI:** 10.1038/s41538-022-00172-0

**Published:** 2022-12-10

**Authors:** Marlana Malerich, Christopher Bryant

**Affiliations:** 1 School of Geosciences, University of Edinburgh, Edinburgh, UK; 2 Department of Psychology, University of Bath, Bath, UK

**Keywords:** Technology, Science, technology and society

## Abstract

Cell-cultivated meat and seafood is getting closer to a reality for consumers in the US and around the world. However, regulators are still largely lagging behind on regulating production and labelling of these products. In a large experimental study using a representative US sample (*N* = 2653), we tested 9 different names for 3 different types of meat and seafood products in terms of their clarity, consumer appeal, and communication of safety and allergenicity. We found that terms proposed by the conventional meat and seafood industry including ‘artificial’ and ‘lab-grown’ tended to score low in terms of consumer appeal, purchase intent, and perceived safety, while ‘artificial’ also had the lowest score on clarity and communicating allergenicity. On the other hand, terms proposed by the cell-cultivated industry including ‘Novari’ scored high in terms of appeal and purchase intent but scored low in terms of clarity. The terms ‘cell-cultured’ and ‘cell-cultivated’ were the best all round labels in terms of clarity, appeal, and communicating safety and allergenicity – in particular, the addition of the prefix ‘cell-’ increased understanding compared to ‘cultured’ or ‘cultivated’ labels. The most-understood label was a short descriptive phrase (‘grown from [animal] cells, not farmed [or fished]’), suggesting that additional wording on packaging could aid consumer understanding in this early stage. A high proportion of consumers were uncertain about the allergen status of cell-cultivated products under all names, suggesting that cell-cultivated products should be labelled as the type of meat they are, and carry applicable allergen information.

## Introduction

The cultivated protein industry has received growing attention in recent years due to rising investment, technological advancement, and progress towards reaching price parity with conventional animal protein. Cultivated protein aims to address the negative externalities increasingly associated with conventional, intensive animal agriculture — including global environmental degradation, dangers to human health, biodiversity loss, and moral concerns.

Global emissions from livestock production contribute over 14% of global greenhouse gases^1^, while the fishing industry represents 4% of global food emissions^2^. Research demonstrates that cultivated meat may combat these consequences, with cultivated chicken, pork, and beef potentially reducing GHG emissions by 17%, 52%, and 85–92%, respectively, compared to their conventionally produced counterparts^3^.

Human health is directly impacted by conventional livestock production, with over 36% of emerging zoonotic diseases associated with animals intended for human consumption^4^. These zoonotic diseases significantly impact human health, livelihood, and, as revealed in the Covid-19 pandemic, global economic resilience^5–7^. Microplastics and mercury in seafood present credible threats to public health^8–12^. The use of prophylactic antibiotics in animal agriculture and aquaculture also exacerbates antimicrobial resistance, again presenting a threat to public health^13–16^. Cultivated proteins are produced in a controlled environment, which may significantly reduce the risk of transmission of zoonotic diseases, remove the need for antibiotics, and minimize the potential of microplastic and other foreign contaminants.

Livestock production and the global fishing industry directly contribute to biodiversity loss, with livestock production accounting for an estimated 30% of human-induced biodiversity loss^17–19^. Aquatic populations have seen a steep decline over the last few decades affecting biodiversity and leading to the breakdown of aquatic ecosystems^20,21^. Cultivated protein production is estimated to utilize 95% less land and 78% less water than conventionally produced meat, significantly reducing the impact on biodiversity^3^ above^3^.

Finally, profound ethical issues are associated with conventional livestock, commercial fishing, and aquaculture. Vast fishing vessels decimate fish populations, which coastal subsistence communities depend upon for food^22^. Recorded human rights infringements and modern slavery are well-documented in the industrialized fishing industry^23^ and intensive animal production^24^. These industries also, of course, harm the animals in question.

Despite well-documented negative externalities, the conventional protein industry plays a vital role in the global economy and food security. Livestock contributes to 17% of global kilocalorie consumption^25^ while fishing and aquaculture support 60 million jobs and 3 billion rely on fish as their primary protein intake^26^. Despite a shift toward alternative proteins, projections indicate global animal protein consumption will increase in the next decade by 14% by 2030 (compared to base period average of 2018–2020)^27^ exacerbating the existing externalities associated with conventional protein production^28,29^.

As cultivated protein reaches the global market, these novel protein products will exist alongside conventional animal products. Livestock and fishing interest groups and associations have expressed reasonable concern about enabling consumers to distinguish cultivated protein from traditional proteins. At the core of the discussion is nomenclature. To date, stakeholders have struggled to reach a consensus on what to call these products^30–32^. The terminology debate centers around consumer appeal and understanding within the cultivated protein industry. The traditional protein industry expresses similar considerations, with the additional requirement that nomenclature refrains from potentially condemning terms such as “clean”^33^. The adoption of a consistent nomenclature is crucial in bringing cultivated protein products to the commercialized market. Accordingly, this paper seeks to add to the nomenclature discussion on meat, chicken, and fish.

Since the introduction of the first cultivated beef burger in 2013, the nomenclature discussion has been distinguished by varying levels of consensus among stakeholders. Precise labeling is essential to commercializing novel foods as consumers must feel comfortable with the product and understand its origins. Beyond consumer acceptance/understanding, consideration must be given to regulatory frameworks, industry preferences, and interest group feedback. Accordingly, there has yet to be stakeholder consensus as reflected in a recent USDA request for information, which resulted in 39 terms put forward^34^.

Early academic and industry leaders referred to the product as “in-vitro”^35,36^, though usage has declined due to consumer associations with artificiality^37^. “Lab-grown” may be less attractive to consumers for potentially similar reasons^38^. The terminology “cultured” is well- utilized^39^ and is relatively well-received by both consumers^38,40,41^ and industry leaders^42^. Good Meat, the only company currently authorized to sell cultivated meat, has used ‘cultured’ in its advertising campaigns^43^. However, “cultured” is associated with aquaculture production and may be misleading in the context of seafood^44^. Consumer studies indicate that consumers responded favorably to “clean meat”^38,45^ and ‘slaughter-free” nomenclature^40^. However, other stakeholders may not consider this nomenclature optimal, specifically conventional animal protein producers^33^. While “cell-based” has been presented as a compromising term supported by industry leaders and organizations such as the North American Meat Institute, it received low consumer appeal ratings^32,44^. The terminology “cultivated” has been increasingly popularized and is now the preferred terminology utilized by entities such as the Good Food Institute^46^. In 2021, industry leader research indicated that 75% of cultivated protein companies preferred “cultivated” terminology^42^.

While preferred terminology is still the subject of debate, products are increasingly making their way to market. In December 2020, Singapore approved the first cultivated meat product for sale^47^, reinforcing the importance of nomenclature consensus in the United States and abroad. In recent years there have been two federal calls for feedback in the United States: (1) the U.S. Food and Drug Administration’s (FDA) 2020 call for feedback on labeling foods comprised of cultivated seafood, and (2) the U.S. Department of Agriculture’s (USDA) 2021 call for comments on the labeling of cultivated meat and poultry.

In October 2020 the FDA issued a Request for Information to solicit feedback to support the naming of cell-cultivated fish products. The U.S. Food and Drug Administration requires that all foods have a label depicting a “common or usual name.” The regulation, 21CFR101.3, requires that the “common or usual name” accurately identifies and describes the product in the most direct and straightforward terms possible to ensure informed customer decisions^48^. In 2020, the FDA called for labelling comments, encouraging discourse on cell-cultivated seafood labelling^49^. Previous research on nomenclature has primarily focused on consumer acceptance rather than identifying a common or usual name that may be regulated by the FDA^38^.

Currently, at least two research papers have examined cultivated seafood nomenclature specifically regarding FDA requirements, both by the same authors. The first study^30^ utilised an online between-subject experiment to test common or usual names using packages of three types of seafood consumers may expect to encounter in the supermarket. In the study, 3186 U.S. adults read the terms “cell-cultured seafood,” “cultivated seafood,” “cell-based seafood,” “cultured seafood,” as well as the phrase, “produced using cellular aquaculture“^30^. The study determined that “cell-based seafood” performed the best out of the names tested concerning clarity, distinctiveness, allergen signaling, and consumer appeal. The second study^31^ built on the first and assessed just two names (“cell-based seafood” and “cell-cultured seafood”) in a sample of 1200 U.S. adults. The authors found that both names enabled most consumers to correctly identify the nature of the products and their potential allergenicity and therefore met the FDA’s key criteria. Interestingly, this study found that the name “cell-based seafood” outperformed “cell-cultured seafood” concerning positivity of overall impressions, positivity of initial thoughts and feelings, and interest in tasting and purchasing. Of the 35 comments the FDA received in response to their feedback request, 24% of the comments the FDA received cited one or both of the Hallman and Hallman studies^50^.

In 2021, the USDA issued a request for information soliciting suggestions for the labeling of cell-cultivated meat and poultry which would address consumer expectations for the products, not be false or misleading, and consider economic data. The agency received an influx of responses. A review of the responses indicates the nomenclature suggestions reflected the lack of consensus across stakeholder groups^34,51^ (Table 1) (Removed nomenclature that had only one suggestion).

**Table 1:** Names suggested by at least two contributors to the USDA call for feedback on labeling cultivated meat and poultry^34^. 158.

Preferred Nomenclature	Percentage of Comments
Cultivated	22%
Cell-Cultured	20%
Lab-Grown	14%
Cell-Based	11%
Cultured	10%
Includes 'Artificial'	8%
Cell-Based Food Product Derived from Meat and Poultry	7%
Cell-Cultivated	5%
Cell-Cultured Food Product	2%

The term ‘cultivated’ had the highest percentage of suggestions and drew support primarily from cell-based meat companies^34^. These results align with research produced by the Good Food Institute in 2021 —identifying that 75% of cell-based meat technology companies preferred the ‘cultivated’ nomenclature^42^. However, some in opposition to the term thought ‘cultivated’ presented “ambiguity” and suggested ‘lab-grown’ as an alternative (a suggestion from a beef industry group)^34^.

‘Cell-cultured’ drew approval from varied interest groups, with traditional protein industry groups such as National Fisheries Institute, National Chicken Council, and Idaho Cattle Association aligning with cultivated technology groups such as BlueNalu and Biomilq^34^. The animal interest group Animal Legal Defence Fund also advocated for this terminology^34^. Nomenclature including “Cultured” had a 20% adoption rate by cultivated meat technology organisations in GFI’s 2021 study^42^.

Traditional meat industry groups/advocates primarily suggested ‘lab-grown’ nomenclature^34^. However, ‘lab-grown’ may prompt negative associations and disgust, as cited in previous consumer-based studies^38^. Therefore, the adoption of “lab-grown” by cell-cultivated meat companies is unlikely. The term “artificial,” also suggested solely by traditional meat industry interest groups^34^, has similar negative connotations and may unintentionally mislead consumers into assuming cultivated protein products do not contain meat. Our consumer survey includes both ‘lab-grown’ and ‘artificial.’

Ultimately, a primary concern of both the cultivated and traditional meat industry is consumer awareness and the ability to differentiate cultivated protein from traditionally produced protein accurately.

This research uses preferred nomenclature identified in recent FDA and USDA feedback requests and consumer survey data to test nomenclature against consumer understanding, appeal, willingness to purchase, and awareness of allergenicity. The present study builds on Hallman and Hallman’s^30,31^ work by presenting consumers with “cell-based” and “cell- cultured” alongside preferred nomenclature gleaned from the USDA’s call for feedback and the addition of a new term, “Novari.” A review of all terms included in our consumer study (Table 2) and the rationale for inclusion is listed below. This is also the first study, to the authors’ knowledge, to test the viability of terms for both cell-cultivated meat and seafood.

**Table 2:** Included nomenclature and rational.

Names Suggested and Included	
Cultivated	‘Cultivated’ received the highest percentage of suggestions in the USDA feedback (22%) (Poinski, 2022), and previous research indicates it is one of the terms preferred by cultivated meat companies (75%) (Keerie, 2021).
Cell-Cultured	'Cell-cultured', proposed by various interest groups, encompassed 20% of overall suggestions (Poinski, 2022). Additionally ‘cell-cultured’ builds upon Hallman and Hallman (2020, 2021) findings and recommendations received in FDA feedback (*Regulations.Gov*, 2021).
Lab-Grown and Includes 'Artificial'	The inclusion of ‘Lab-grown’ and ‘Artificial’ (suggestions by traditional meat producers in the USDA feedback) are included to test feasibility of appeal, purchase likelihood, and perceived safety despite previous studies indicating consumers may be wary of such terms (Bryant & Barnett, 2019)
Cell-Based	Inclusion of ‘cell-based’ builds upon Hallman and Hallman (2020, 2021) findings, and aligns with suggestions received in FDA feedback (*Regulations.Gov*, 2021). Additionally, ‘cell-based’ made up 11% of USDA feedback (Poinski, 2022).
Cultured	‘Cultured’ made up 10% of USDA suggestions and previous research suggests that nomenclature including ‘Cultured’ is preferred by some industry leaders (20%) (Keerie, 2021).
Cell-Cultivated	Cell-cultivated received 5% of USDA suggestions (Poinski, 2022) and is included to test feasibility alongside greater-preferenced nomenclature.
Names Suggested and Not Included	
Cell-Based Food Product Derived from Meat and Poultry Cell-Cultured Food Product	Due to the inclusion of ‘Cell-Based and ‘Cell-Cultured’ and their similarity to these longer-form phrases, these terms (suggestions in USDA feedback) (Poinski, 2022) are not included in our study.
Names not Suggested but Were Included	
Novari	Novari is a coined term (derived from the Latin verb “novo” or “to make new”). Its inclusion in the study intends to assess how a term with no previous obvious connection to meat or seafood source (conventional or cultured) may test the feasibility of appeal, purchase likelihood, and perceived safety.
Descriptive phrase (‘Grown from [animal] cells, not farmed [or fished]’)	The study includes a description of cell-cultivated products with no particular name attached.

## Results

In this section, we present the results of the experiment for the 9 different labels for beef, chicken, and salmon products. Participants were allocated independently and at random to one of 9 labels and one of 3 products, yielding the following sample sizes for each condition (Table 3):

**Table 3:** Number of participants in each of 27 product/label conditions.

	Beef	Chicken	Salmon	TOTAL
Cell-cultivated	105	95	95	295
Cultivated	91	109	96	296
Cell-cultured	96	95	100	291
Cultured	99	97	99	295
Cell-based	113	104	80	297
Novari	97	101	94	292
Lab-grown	87	101	108	296
Artificial	102	87	106	295
Descriptive	92	101	103	296
TOTAL	882	890	881	2653

### Understanding of product

**Fig. 1:**
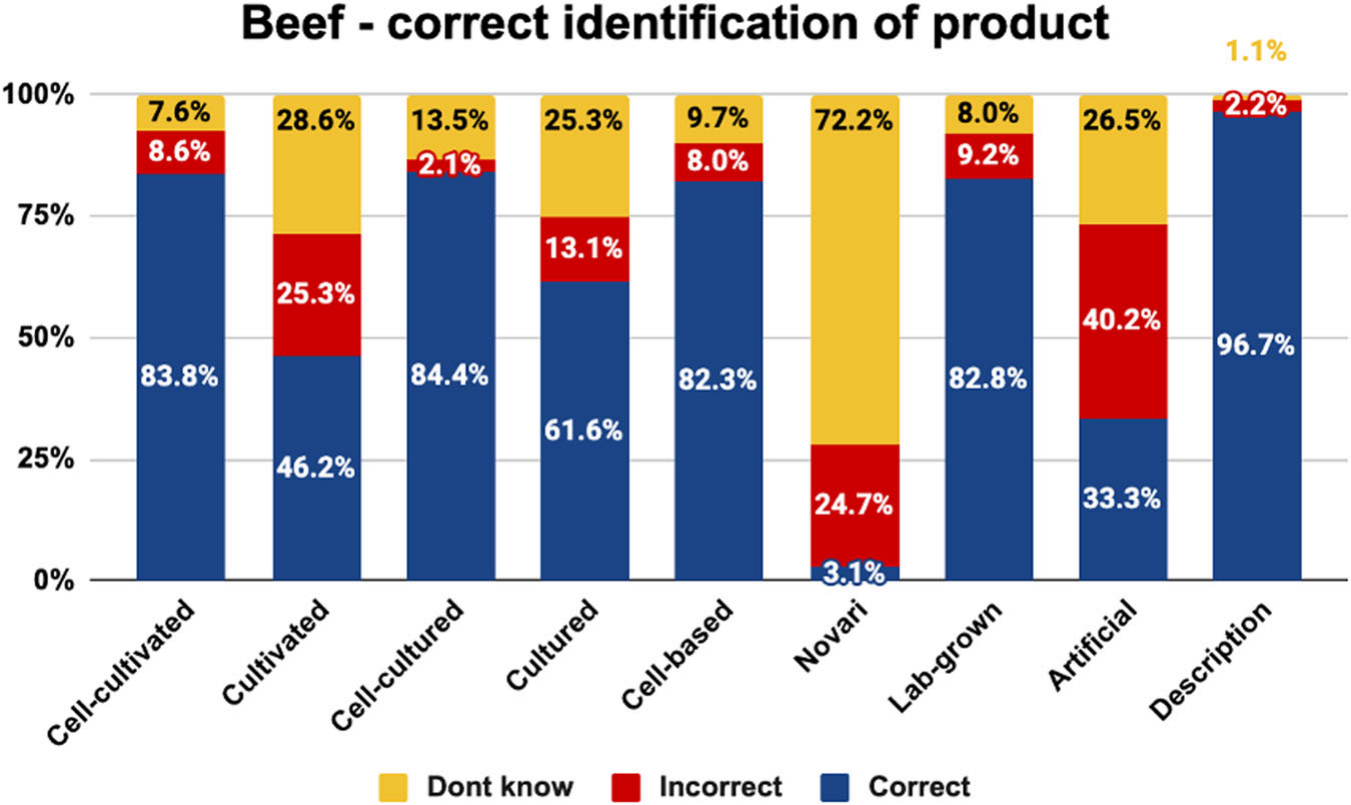
Correct identification of beef by label. The graph shows the proportion of respondents who selected the correct or incorrect cell-cultivated beef product description, or did not know, by experimental condition.

**Fig. 2:**
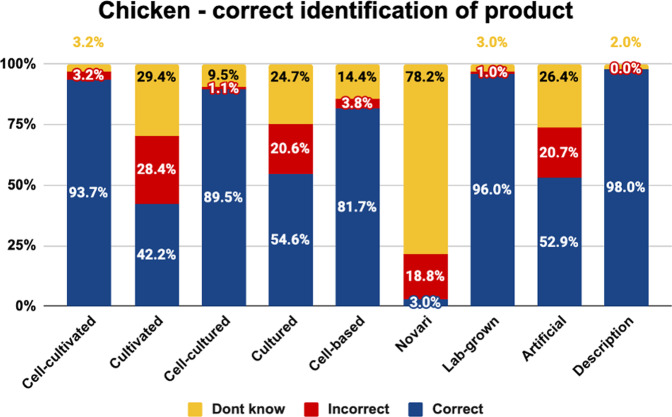
Correct identification of chicken by label. The graph shows the proportion of respondents who selected the correct or incorrect cell-cultivated chicken product description, or did not know, by experimental condition.

**Fig. 3:**
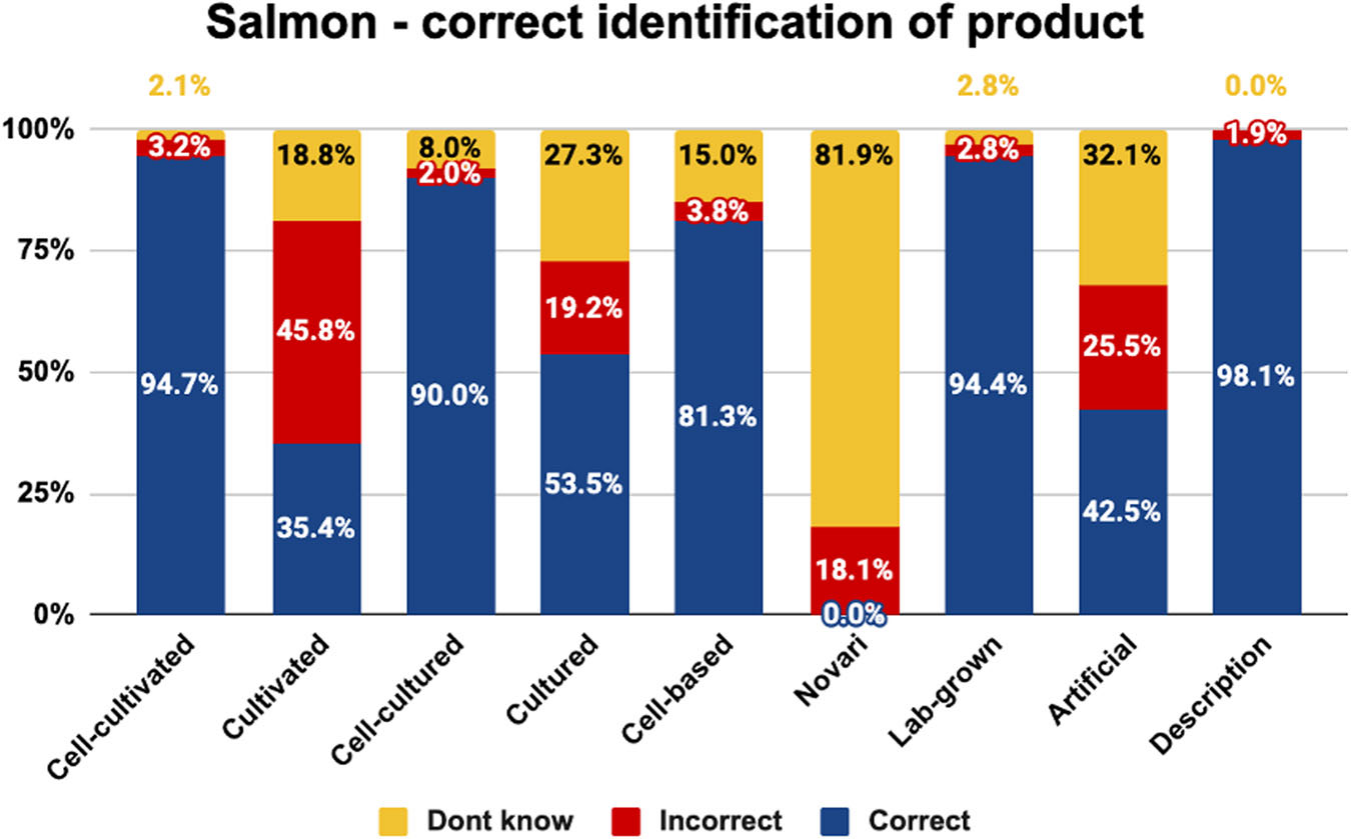
Correct identification of salmon by label. The graph shows the proportion of respondents who selected the correct or incorrect cell-cultivated salmon product description, or did not know, by experimental condition.

Our first set of analyses focused on correct understanding of the different labels. Here, we show the percentage of consumers in each condition identifying the nature of the product correctly, incorrectly, or answering don’t know (Figs. 1–3).

As shown, the highest proportion of consumers correctly identified the nature of cell-cultivated products under the names ‘cell-cultivated’, ‘cell-cultured’, ‘cell-based’, ‘lab grown’, and the descriptive phrase. These labels were correctly understood by more than 80% of consumers for beef, chicken, and salmon.

The labels ‘cultivated’, ‘cultured’, and ‘artificial’ were less well-understood, being correctly identified by 33–62% of consumers for beef, chicken, and salmon. While ‘cultured’ and ‘cultivated’ were incorrectly identified by 13–28% of beef and chicken consumers, this rose to 19–46% of salmon consumers. This is likely due to the conflation of these terms with aquaculture. Moreover, the term ‘artificial’ was misunderstood by 21–40% of beef, chicken, and salmon consumers, and was often incorrectly interpreted as referring to plant-based products.

Finally, the label ‘novari’ was the least well-understood label, being correctly identified by just 0–3% of consumers. While the proportion of people incorrectly understanding this term was relatively low (18–25%), the proportion of consumers who said they did not know what the term referred to was higher than any other term (72–82%). This indicates that the term ‘novari’ was unfamiliar, and did not communicate much information about the nature of the product.

Chi square analyses indicated a significant difference in the proportion of respondents giving each answer for beef (<^2^(16) = 366.844, *p* < 0.001), chicken (<^2^(16) = 429.085, *p* < 0.001), and salmon (<^2^(16) = 514.390, *p* < 0.001).

Pairwise comparisons are indicated in Table 4. Superscript letters indicate that the value differs significantly from the condition labelled with the same letter in the leftmost column.

**Table 4:** Pairwise comparisons between names on the percentage choosing the correct definition.

	BEEF - Correct definition	CHICKEN - Correct definition	SALMON - Correct definition
**Cell-cultivated**^**a**^	83.8%^b,d,f,h^	93.7%^b,d,f,h^	94.7%^b,d,f,h^
**Cultivated**^**b**^	46.2%^a,c,e,f,g,i^	42.2%^a,c,e,f,g,i^	35.4%^a,c,e,f,g,i^
**Cell-cultured**^**c**^	84.4%^b,d,f,h^	89.5%^b,d,f,h^	9.0%^b,d,f,h^
**Cultured**^**d**^	61.6%^a,c,e,f,h,i^	54.6%^a,c,e,f,g,i^	53.5%^a,c,e,f,g,i^
**Cell-based**^**e**^	82.3%^b,d,f,h,i^	81.7%^b,d,f,g,h,i^	81.3%^b,d,f,h,i^
**Novari**^**f**^	3.1%^a,b,c,d,e,g,h,i^	3.0%^a,b,c,d,e,g,h,i^	0.0%^a,b,c,d,e,g,h,i^
**Lab-grown**^**g**^	82.8%^b,f,h^	96.0%^b,d,e,f,h^	94.4%^b,d,f,h^
**Artificial**^**h**^	33.3%^a,c,d,e,f,g,h^	52.9%^a,c,e,f,g,i^	42.5%^a,c,e,f,g,i^
**Control**^**i**^	96.7%^b,d,e,f,h^	98.0%^b,d,e,f,h^	98.1%^b,d,e,f,h^

### Appeal, purchase intent, and perceived safety

**Fig. 4:**
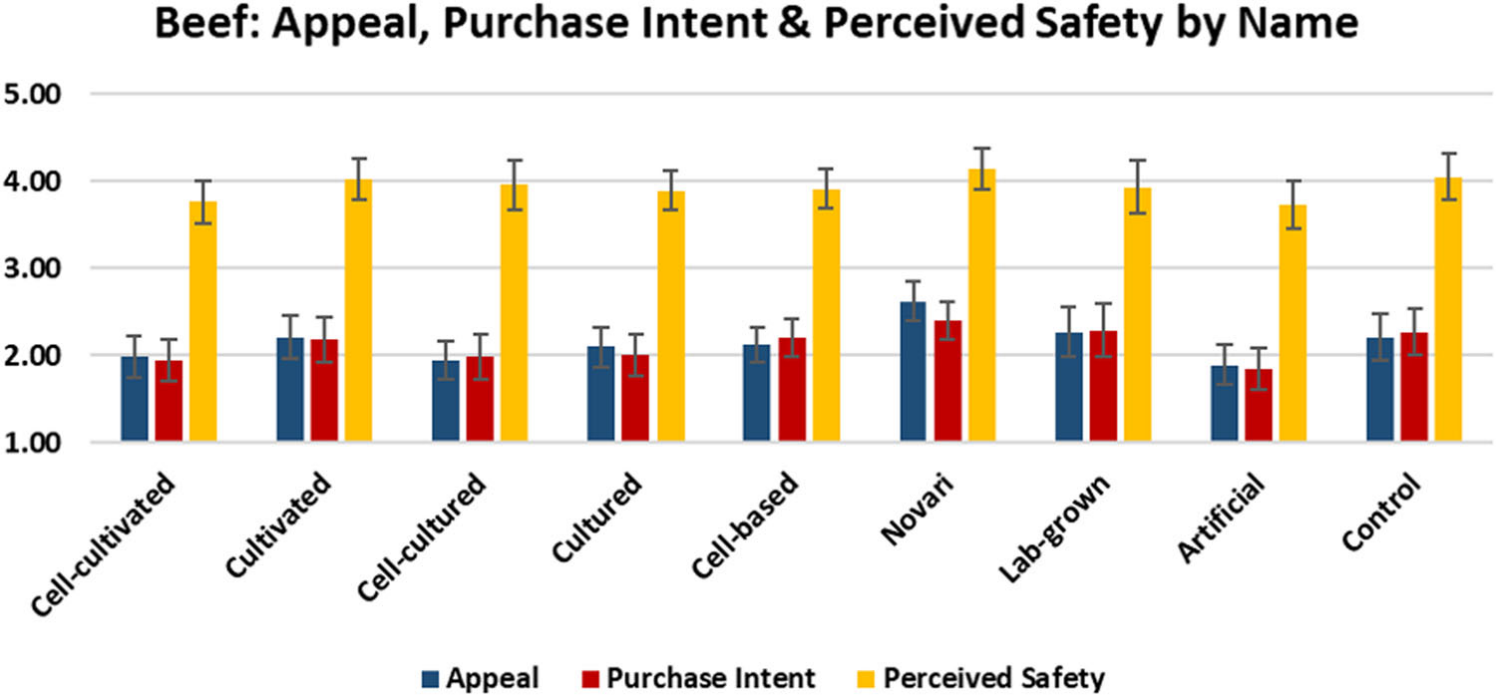
Appeal, purchase intent, and perceived safety of beef by label. The graph shows the mean scores with 95% confidence intervals for the subjective appeal, purchase intent, and perceived product safety of cell-cultivated beef products by experimental condition.

**Fig. 5:**
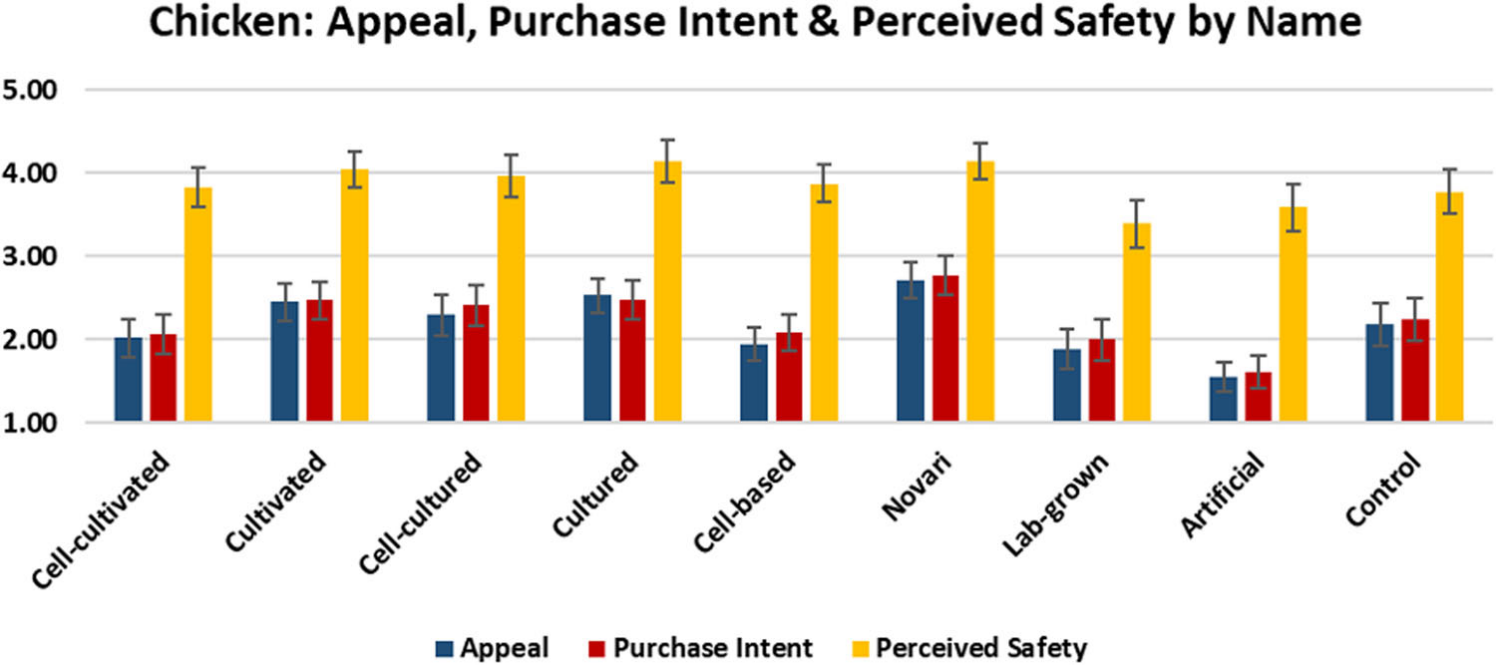
Appeal, purchase intent, and perceived safety of chicken by label. The graph shows the mean scores with 95% confidence intervals for the subjective appeal, purchase intent, and perceived product safety of cell-cultivated chicken products by experimental condition.

**Fig. 6:**
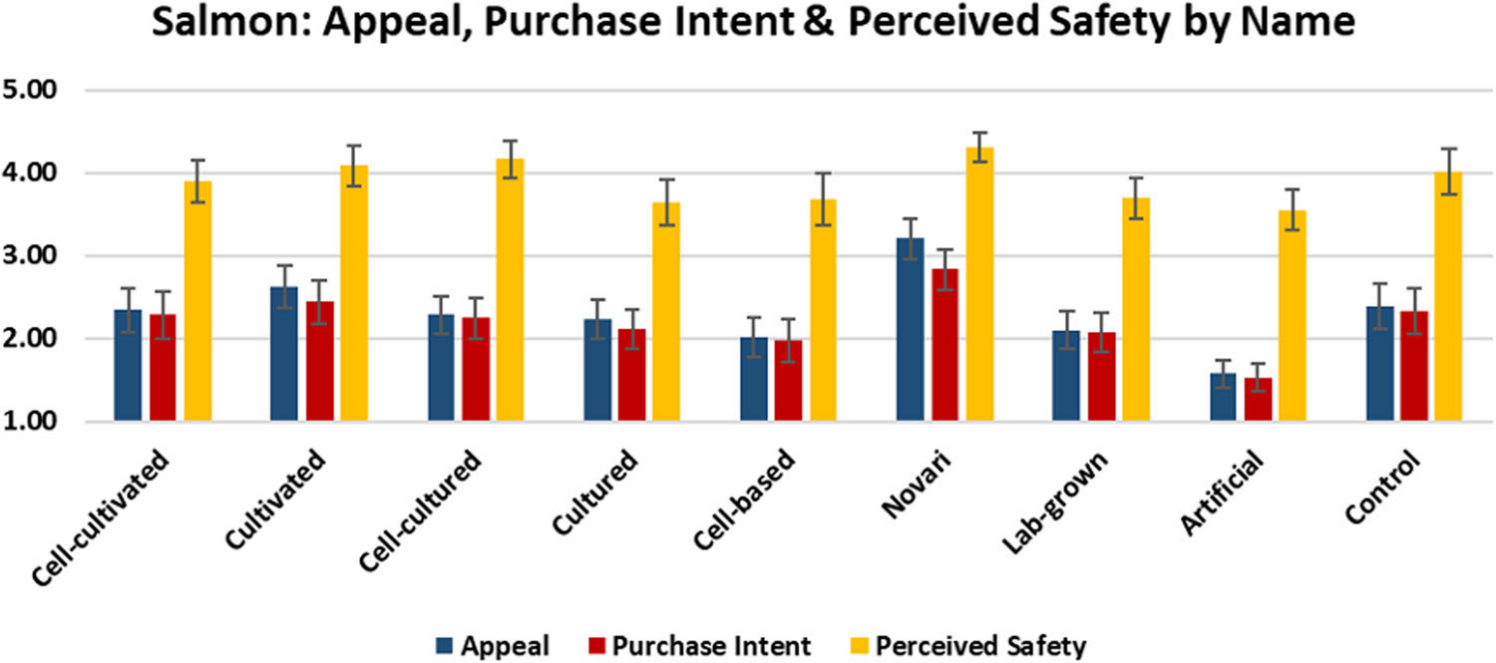
Appeal, purchase intent, and perceived safety of salmon by label. The graph shows the mean scores with 95% confidence intervals for the subjective appeal, purchase intent, and perceived product safety of cell-cultivated salmon products by experimental condition.

In this section, we report the mean scores for each product’s appeal, purchase likelihood, and perceived safety (understanding of risk for individuals who are not allergic to the given type of meat) (Figs. 4–6).

As shown, the term ‘novari’ was associated with the highest levels of appeal, purchase intent, and perceived safety for beef, chicken, and salmon. Conversely, the term ‘artificial’ was associated with the lowest levels of appeal and purchase intent for beef, chicken, and salmon, and the lowest level of perceived safety for beef and salmon (for chicken, ‘lab-grown’ was associated with the lowest level of perceived safety).

For beef and salmon, ‘cultivated’ was perceived more positively than ‘cultured’ and ‘cell-based’, whereas for chicken, ‘cultured’ was perceived slightly more positively than ‘cultivated’. Generally, adding the prefix ‘cell-’ to the terms ‘cultured’ and ‘cultivated’ increased understanding, but decreased consumer appeal and perceived safety.

ANOVA analyses indicated that there were significant differences between labels in appeal for beef (F(8,873) = 3.348 *p* = 0.001), chicken (F(8,881) = 10.193 *p* < 0.001), and salmon (F(8,872) = 13.742 *p* < 0.001).

There were also significant differences between labels in purchase likelihood for beef (F(8,873) = 2.256 *p* = 0.022), chicken (F(8,881) = 8.084 *p* < 0.001), and salmon (F(8,872) = 8.297 *p* < 0.001).

Finally, there were significant differences between labels in perceived safety for chicken (F(8,736) = 4.113 *p* < 0.001) and salmon (F(8,748) = 4.337 *p* < 0.001), but not for beef (F(8,738) = 1.100 *p* = 0.361).

Pairwise comparisons are indicated in Table 5. Superscript letters indicate that the value differs significantly from the condition labelled with the same letter in the leftmost column.

**Table 5:** Pairwise comparisons between names for appeal, purchase intent, and perceived safety.

	BEEF			CHICKEN			SALMON		
	Appeal	Purchase intent	Perceived safety	Appeal	Purchase intent	Perceived safety	Appeal	Purchase intent	Perceived safety
Cell-cultivated^a^	1.99^f^	1.94	3.76	2.02^d,f^	2.06^f^	3.82	2.34^f,h^	2.28^h^	3.90
Cultivated^b^	2.21	2.18	4.03	2.45^b,g,h^	2.47^h^	4.04^g^	2.63^f,g,e,h^	2.44^h^	4.08
Cell-cultured^c^	1.95^f^	1.99	3.96	2.29^h^	2.41^h^	3.96^g^	2.28^f,h^	2.24^f,h^	4.16^h^
Cultured^d^	2.10	2.01	3.90	2.53^a,b,g,h^	2.48^h^	4.13^g^	2.23^f,h^	2.11^f,h^	3.64^f^
Cell-based^e^	2.13	2.20	3.92	1.95^b,d,f^	2.08^f^	3.87	2.01^b,f^	1.98^f^	3.68^f^
Novari^f^	2.62^a,c,h^	2.40	4.15^h^	2.71^a,b,g,h,i,^	2.77^a,b,g,h,i^	4.13^g^	3.20^a,b,c,d,e,g,h,i^	2.83^c,d,e,g,h^	4.31^c,d,e,g^
Lab-grown^g^	2.26	2.29	3.93	1.89^b,d,f^	2.00^f^	3.39^b,c,d,f^	2.10^b,f,h^	2.07^f,h^	3.70^f^
Artificial^h^	1.89^f^	1.85^f^	3.73	1.55^b,c,d,f,i^	1.61^b,c,d,f,i^	3.58	1.58^a,b,c,d,f,g,h,i^	1.53^f^	3.55^c,f^
Descriptive^i^	2.21	2.27	4.05	2.18^f,h^	2.24^f,h^	3.78	2.39^f,h^	2.33^h^	4.01

Malerich & BryantNomenclature Study

### Perceived safety for allergy-sufferers

**Fig. 7:**
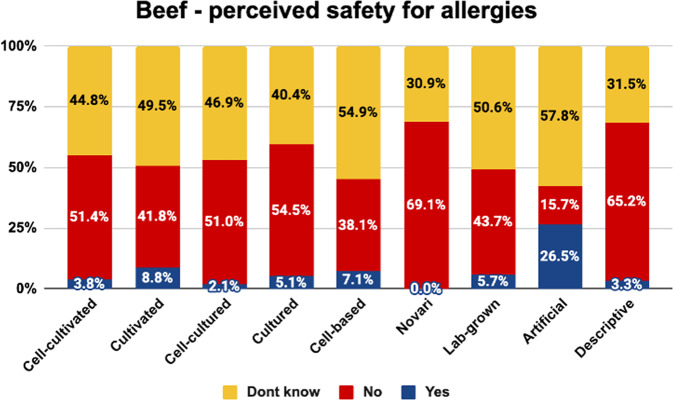
Perceived allergen safety of beef by label. The graph shows the proportion of people who correctly identified that cell-cultivated beef products are not safe for allergy-sufferers, incorrectly identified that they are safe for allergy sufferers, and did not know.

**Fig. 8:**
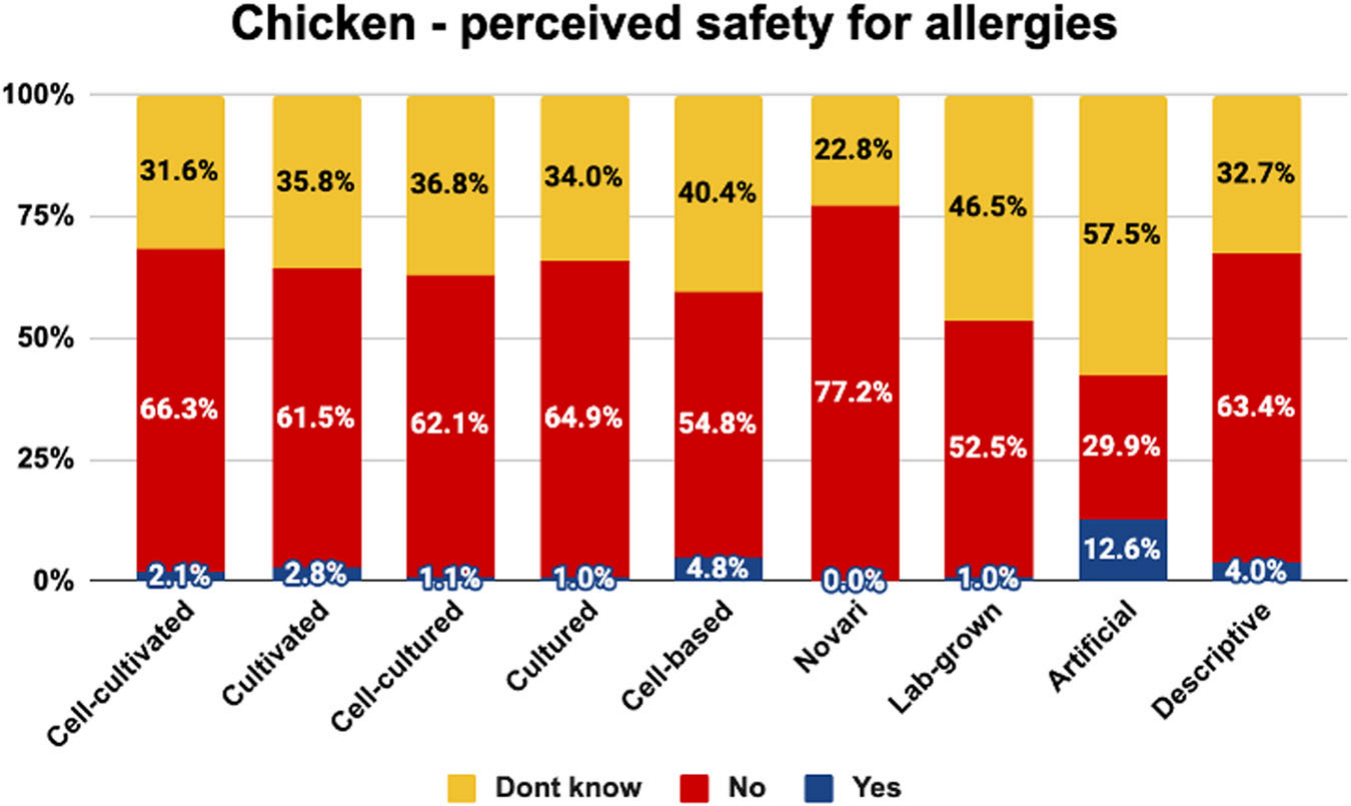
Perceived allergen safety of chicken by label. The graph shows the proportion of people who correctly identified that cell-cultivated chicken products are not safe for allergy-sufferers, incorrectly identified that they are safe for allergy sufferers, and did not know.

**Fig. 9:**
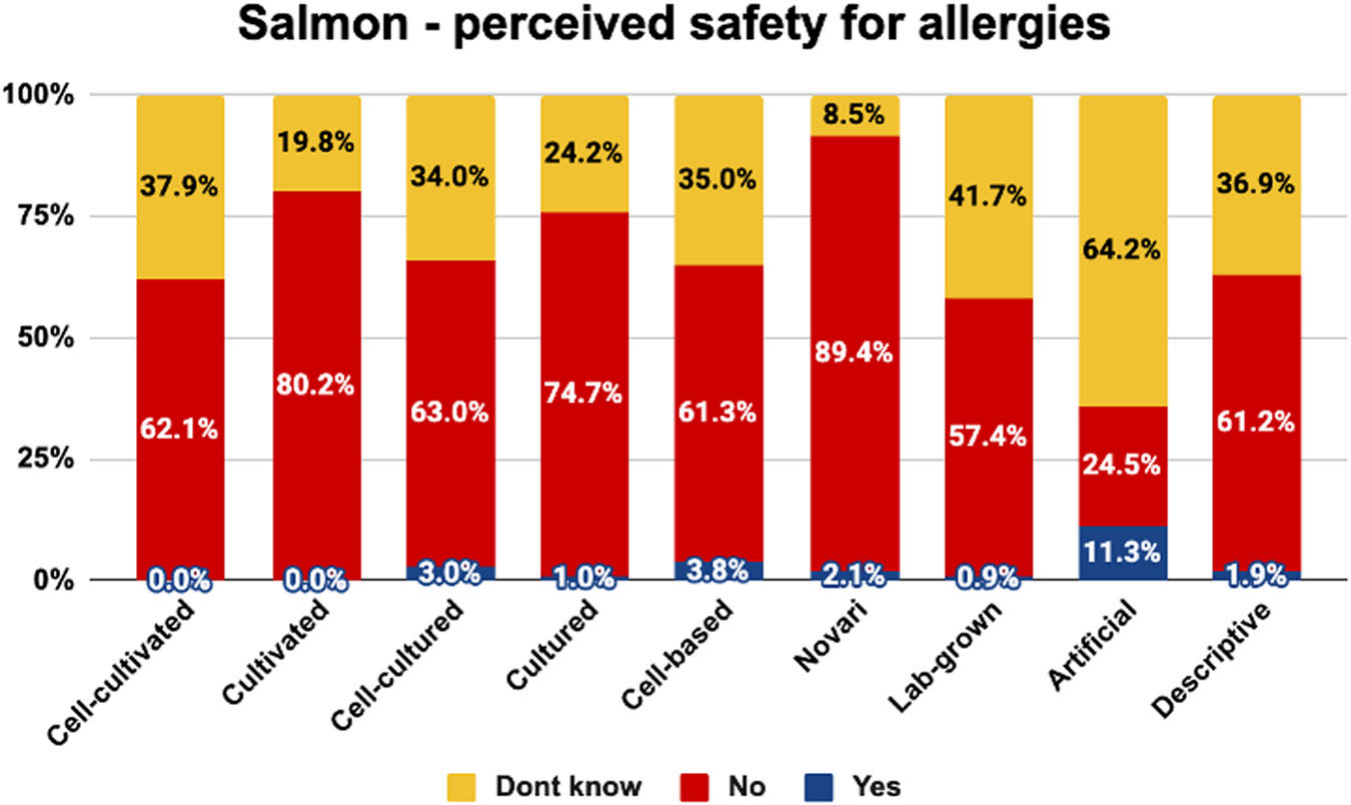
Perceived allergen safety of salmon by label. The graph shows the proportion of people who correctly identified that cell-cultivated salmon products are not safe for allergy-sufferers, incorrectly identified that they are safe for allergy sufferers, and did not know.

Here, we report on the percentage of people who thought that products under each label were safe for people who are usually allergic to the given type of meat (Figs. 7–9).

As shown, the term ‘novari’ was generally associated with the highest proportion of consumers correctly identifying that the product was not suitable for allergy-sufferers. Between 69–89% identified products with this label as unsafe for allergy-sufferers, while 0–2% incorrectly said it would be safe.

The term ‘artificial’ was associated with the highest proportion of consumers incorrectly saying that the products would be safe for allergy-sufferers (11–27%), the lowest proportion of consumers correctly saying that the products would not be safe for allergy- sufferers (16–30%) and the highest proportion of consumers being uncertain about the allergy safety status (58–64%). This is likely due to the misleading nature of this label indicating that the products are not beef, chicken, and salmon, respectively, and thus causing consumers to infer that they would be safe for people who are usually allergic to those products.

The proportion of consumers who correctly identified products as unsuitable for allergy-sufferers was similar for the other labels for most products. The terms ‘cell-cultivated’, ‘cultivated’, ‘cell-cultured’, ‘cultured’, ‘cell-based’, ‘lab-grown’ and the descriptive phrase were correctly perceived as unsafe for allergy-sufferers by 38–66% of consumers who saw beef or chicken. The terms ‘cultivated’ and ‘cultured’ were perceived as unsafe for allergy- sufferers by 75–80% of those who saw salmon products, presumably because they were more often misunderstood as referring to aquaculture-produced salmon. Indeed, the overall rate of people identifying salmon products as unsafe for allergy-sufferers was higher, presumably because seafood allergies are far more common than chicken/beef allergies.

Chi square analyses indicated that the proportion of consumers in each category differed significantly between naming conditions for beef (<^2^(16) = 127.036, *p* < 0.001), chicken (<^2^(16) = 74.389, *p* < 0.001), and salmon (<^2^(16) = 136.693, *p* < 0.001).

Pairwise comparisons are indicated in Table 6. Superscript letters indicate that the value differs significantly from the condition labelled with the same letter in the leftmost column.

**Table 6:** Pairwise comparisons between names on the percentage correctly identifying products as non-allergy-safe.

	BEEF - Correct definition	CHICKEN - Correct definition	SALMON - Correct definition
Cell-cultivated^a^	51.4%^h^	66.3%^h^	62.1%^f,h^
Cultivated^b^	41.8%^f,h^	61.5%^h^	80.2%^g,h^
Cell-cultured^c^	51.0%^h^	62.1%^h^	63.0%^f,h^
Cultured^d^	54.5%^h^	64.9%^h^	74.7%^h^
Cell-based^e^	38.1%^f,h,i^	54.8%^f,h^	61.3%^f,h^
Novari^f^	69.1%^b,e,g,h^	77.2%^e,g,h^	89.4%^a,c,e,g,h,i^
Lab-grown^g^	43.7%^f,h^	52.5%^f^	57.4%^b,f,h^
Artificial^h^	15.7%^a,b,c,d,e,f,g,i^	29.9%^a,b,c,d,e,f,i^	24.5%^a,b,c,d,e,f,g,i^
Descriptive^i^	65.2%^e,h^	63.4%^h^	61.2%^f,h^

### Comparing types of meat

**Fig. 10:**
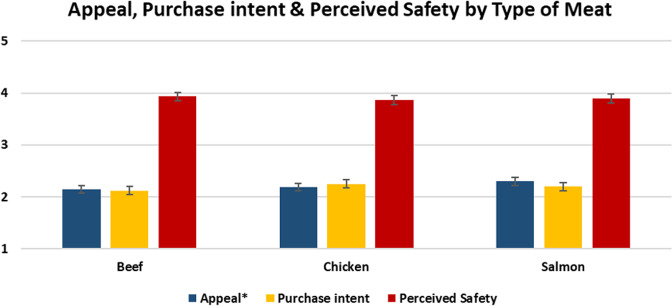
Appeal, purchase intent, and perceived safety by product type. The graph shows the mean scores with 95% confidence intervals for the subjective appeal, purchase intent, and perceived product safety of cell-cultivated meat across experimental conditions by type of meat.

We employed an additional ANOVA analysis to compare purchase intent across labels between the three different types of meat presented. The results are shown in Fig. 10.

The ANOVA analysis found no significant differences in purchase intent (F(2,2650) = 2.292, *p* = 0.101) or perceived safety (F(2)2246 = 0.647, *p* = 0.524) between the three types of meat. However, there was a significant difference between the types of meat in terms of appeal (F(2)2650 = 3.852, *p* = 0.021) – in particular, salmon was rated as significantly more appealing than beef. For beef, 20% said they were somewhat or extremely likely to purchase; for chicken, 21.8% said they were somewhat or extremely likely to purchase; for salmon, 21.9% said they were somewhat or extremely likely to purchase.

It is noteworthy that these findings do not mirror those of previous studies, which have found cultivated beef to be more appealing than cultivated chicken, which was in turn found to be more appealing than cultivated fish^52^. It is also worth noting that these purchase intention rates are somewhat lower than those observed in previous studies; this may be due to the small amount of information given to participants, and due to the high inclusion of unappealing names.

### Demographic predictors of purchase intent

We also employed multiple linear regression to investigate the demographic factors associated with purchase cell-cultivated meat and seafood purchase intent. The results are shown in Table 7. Note that, for categorical variables, the reference values are Gender=Male and Region=Northeast.

**Table 7:** Multiple linear regression showing significant demographic predictors of cultivated meat and seafood purchase intent.

F(7,2603) = 17.08, *p* < 0.001 R^2^ = 0.044, Adj R^2^ = 0.041					
	Unst. Beta	Std. Error	Std. Beta	t	*p*
(Constant)	2.812	0.103	−	27.411	<0.001
Age*	−0.008	0.001	−0.112	−5.807	<0.001
Gender: Female*	−0.402	0.048	−0.163	−8.43	<0.001
Gender: Other	0.396	0.268	0.029	1.479	0.139
Income	−0.002	0.014	−0.003	−0.169	0.866
Region: South	−0.045	0.065	−0.018	−0.699	0.484
Region: Midwest	−0.08	0.075	−0.026	−1.072	0.284
Region: West	0.035	0.077	0.011	0.456	0.648

* *denotes predictors which are significant at p<0.05*.

As shown, the regression identified two significant predictors of cell-cultivated meat and seafood purchase intent. Younger respondents were more likely to purchase cell-cultivated meat and seafood, whereas females were significantly less likely to purchase than males.

## Discussion

In this study, we gathered the commonly-suggested names for cultivated meat to FDA and USDA requests for information, and tested them experimentally among a representative sample of US consumers to determine their clarity, consumer appeal, and communicativeness of safety.

One notable result observed here is that the short descriptive phrase achieved the greatest degree of understanding compared to all tested terms. Though there was no significant difference from the terms ‘cell-cultivated’, ‘cell-cultured’, and ‘lab-grown’, the descriptive phrase was the only term to achieve above 95% understanding across all types of meat tested. This may suggest that, at this stage of industry development, full descriptions are likely to be more informative than any labels for these unfamiliar products.

Names which invoke science and technology tended to have lower measures of appeal and purchase intent. This concurs with previous findings, which found that immediate associations were important in explaining differences in willingness to eat cultivated meat under different labels^38^. The label ‘lab-grown’, while generally well-understood, also tended to be associated with lower levels of consumer appeal and purchase intent. It was also associated with a higher level of uncertainty about the safety for allergic consumers.

The findings indicate that, while some terms were unclear, the addition of the prefix ‘cell-’ to ‘cultured’ or ‘cultivated’ improved clarity when describing these products. Previous research has indicated that the imagery of cultivation is a positive one in this context^40^ and this term is now associated with other industry verbiage (e.g. the process of cultivating meat, using cultivators to grow meat). Therefore, based on these data, the terms ‘cell-cultured’ or ‘cell- cultivated’ appear to be the most appropriate terms due to a high level of understanding, a relatively high level of consumer appeal and purchase intent, and a relatively high understanding that such products are not safe for allergy-sufferers.

It is also notable that there was a high degree of misunderstanding as to the allergenicity of cell-cultivated meat and seafood products, even for terms which were well-understood including the descriptive phrase. This misconception was especially high for products labelled as ‘artificial’, while it was lower for salmon products, for which allergies are more common compared to chicken and beef. Therefore, it is important that cell-cultivated meat and seafood products should be labelled as containing meat and seafood and should carry appropriate allergen warnings regardless of nomenclature.

This research adds to the discussion of Hallman and Hallman^30,31^, who assessed common or usual names for cell-cultivated seafood in their first study. Though they did not test the term ‘cell-cultivated’, they did find that the term ‘cell-cultured’ and the phrase ‘cultivated from the cells of’ were both seen as clear and appropriate, yet relatively unappealing. Ultimately, the study determined that ‘cell-based’ performed the best out of the names tested concerning distinctiveness, allergen signaling, and consumer appeal. The second study built on the first and assessed two names (‘cell-based’ and ‘cell-cultured’), finding that ‘cell-based’ outperformed ‘cell-cultured’. Our findings do not concur here – we find that ‘cell-cultured’ and ‘cell-cultivated’ tend to achieve the best levels of understanding, appeal, and clarity regarding allergenicity overall.

These findings have implications for manufacturers of cell-cultivated meat and seafood products and for regulators. In the United States, the FDA (21CFR102.5) requires a ‘common or usual name’ which ‘shall accurately identify or describe, in as simple and direct terms as possible, the basic nature of the food or its characterizing properties or ingredients’^48^. Therefore, a consumer’s ability to easily and accurately identify a product is crucial in establishing a common or usual name.

Cell-cultivated meat may be comparable in most respects (allergen status, nutritional content, taste, etc.) to conventionally produced meat. The distinguishing factor, or ‘characterizing property’, is the production method (rather than the ingredients) and the product name should therefore reflect this. Among the names tested in this study, ‘cell-cultivated’ had the optimal balance of achieving both clarity of product ingredients and consumer appeal. In addition to clarity of product and production, consumers must also be aware of allergens. In our study, consumers were uncertain about the product’s allergen status, and therefore, in the interest of food safety, cell-cultivated meat and seafood ought to be appropriately labeled with meat and seafood allergen information. The FDA requires labeling on all packaged allergen-containing foods, including single-ingredient products such as packaged meat and fish^53^. Therefore, cell- cultivated meat and seafood, which will contain allergens, will likely be required to carry an allergen-signaling label. Furthermore, categorizing cell-cultivated meat/seafood as something other than meat/seafood could cause consumers significant confusion with respect to allergenicity.

Our analysis indicated no clear consensus on an established common or usual name for cell-cultivated products. As cell-cultivated products gain market prominence, consumer awareness is likely to rise — leading to a better understanding of the products and the emergence of a distinct common or usual name that allows consumers to distinguish between cell-cultivated and conventionally produced products. However, for now, general public awareness of cell-cultivated meat and seafood and related technology is minimal. Accordingly, our research illustrates that a descriptive phrase was more successful in consumers accurately identifying the product than any of the exploratory terms. While such phrases are likely too long to be used in the nomenclature per se, their use on packaging alongside a product name could improve understanding and appeal.

There are some key limitations to be acknowledged in this study. First, self-reported online survey results are imperfect in terms of validity and reliability. While findings are therefore unlikely to be perfectly replicated in real food purchase decision situations, we did take some measures to increase validity, such as using attention checks to exclude poor quality responses and using realistic images of product packaging. Second, the Prolific survey platform acknowledges that participants will be subject to some selection bias based on response time, interest, study pricing, and other factors^54^. However, we attempted to mitigate these biases by using a large nationally-representative sample, and employing a robust series of quality and attention checks.

Future research could investigate the possibility of an as-yet-undiscovered term for cell- cultivated meat and seafood which is both appealing and intuitive. We tested the novel term ‘Novari’ in this study and found that it achieved the former but not the latter. Research could also explore which specific forms of regulatory approval and on-package labels would be most reassuring to consumers who are considering trying cell-cultivated meat and seafood. Social research could also explore the types of cell-cultivated meat and seafood dishes consumers will find most appealing, in order to guide product development. Another promising area for future research is the impact of additional information about products and processing on purchase intent — in this study, very little information was given by design. Finally, future studies could assess the interaction between a combination of a proposed common or usual name and a short descriptive phrase - i.e. the descriptive phrase could supplement understanding alongside a name.

As we have argued in this paper, widespread adoption of these products could yield significant benefits for the planet. Cell-cultivated meat and seafood offer a substitute for conventional meat and seafood consumption, potentially mitigating many negative impacts of the conventional meat and seafood industries. Consumers are more aware than ever of food choice implications and have expressed a willingness to transition to more sustainable options, including cell-cultivated options^55^.

Cell-cultivated meat and seafood companies in the United States and globally have successfully produced a variety of prototype products — pending regulatory approval. A recent multinational study indicates that regulatory feedback may increase consumer acceptance of cultivated meat^56^ reinforcing the necessity for regulatory frameworks to embrace a cohesive narrative around these novel products, including naming. Regulatory approval in one nation may influence other nations, as Singaporean regulation of cell- cultivated chicken in 2020 incited speculation about regulatory approval in other countries. Currently, there are pathways for regulatory approval in nations across the globe and the European Union^42^. Additionally, with cross-national partnerships such as those between cultivated meat company Aleph Farms (Israel) with BRF (Brazil) and Mitsubishi (Japan), nomenclature cohesivity may be required for packaging and marketing, incentivizing expedited regulatory decisions in multiple countries at once^57,58^. Industry leaders, investors, and policymakers suggest a 1–5 year timeline for wide-scale market availability^59^. Thus, there is an urgency to address regulatory issues (such as adopting a common or usual name) as regulations may lag behind consumer demand and industry innovation. If we achieve the necessary advancements in technology and regulation, cell-cultivated meat and seafood can secure great improvements in animal treatment, environmental outcomes, and public health.

## Methods

To test the feasibility of a range of different names, we conducted an online experimental survey with a representative sample of US consumers. This study received ethical approval from the University of Bath Psychology Research Ethics Committee (PREC 22–100). Respondents provided written informed consent to take part in the study.

### Participants

Participants were recruited through the online research platform, Prolific, and were each paid $1.00 for their participation in a 5-minute survey. We tested 9 different labels for each of 3 different products, yielding a total of 27 unique experimental conditions. In order to ensure sufficient power for comparisons between all conditions, we recruited 2700 participants. We recruited a total of 2,708 participants, and removed 55 of them, yielding a final sample of 2653 participants. Participants were removed for various reasons, including unfinished responses, speeding, not giving consent, failing attention check questions, and being under the age of 18.

**Fig. 11:**
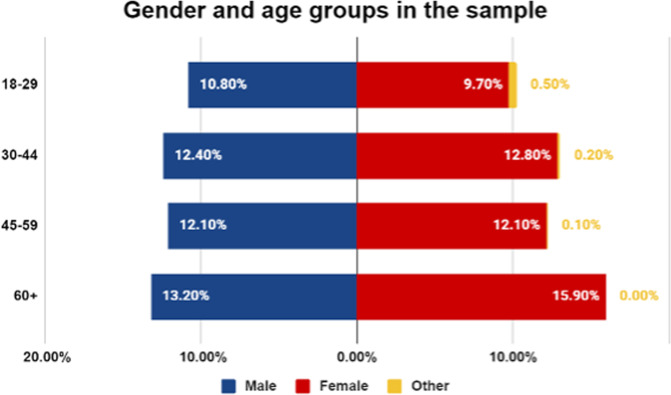
The proportion of participants in each gender and age group category. As shown, we recruited a representative sample based on age/gender groups.

We aimed to recruit a representative sample of US consumers, and to this end, we employed a stratified sampling approach, recruiting proportional numbers of males and females in the age brackets 18–29, 30–44, 44–59, and 60+. The proportion of participants belonging to each age/gender group is represented in Fig. 11:

### Procedure

Participants were recruited through Prolific and directed to the survey, which was hosted on Qualtrics. First, participants read information about the study and gave their consent to take part. After completing a CAPTCHA to verify human responses, they were randomly allocated to one of 3 types of meat, and one of 9 product labels (see Table 8).

**Table 8:** Three types of meat and nine labels used in the study.

Types of meat	Labels
Beef	Cell-cultivated
Chicken	Cultivated
Salmon	Cell-cultured
	Cultured
	Cell-based
	Novari
	Lab-grown
	Artificial
	Descriptive phrase (‘Grown from [animal] cells, not farmed [or fished]’)

They saw an image of food packaging corresponding to the labels above, and answered a series of questions about the product they saw. They were asked to identify what the product was based on what the packaging communicated about the product — whether it was hunted/fished in the wild, farm raised, produced by animal cells in a food facility, or was plant-based. They could also indicate that they don’t know.

Participants were also asked how likely they would be to purchase the product (5 point scale; Extremely unlikely – Extremely likely), and how appealing they found the product (5 point scale; Not at all appealing – Very appealing). Next, they were asked whether it would be safe to eat the product for someone who was allergic to the meat usually (Yes, No, Don’t know), and how safe it would be to eat for someone who was not allergic to the meat usually (5 point scale; Very unsafe – Very safe, or Don’t know).

In the last section of the survey, participants answered demographic questions about themselves, including gender, age, income, and US region. There were also 2 quality control questions throughout the survey: in one question, participants had to choose ‘I am a human and I am paying attention’ from a list of four options, and in the second question, they had to choose ‘Yellow’ from a list of five colours. Those who failed either attention check were removed from the survey. Finally, participants were thanked for their participation, debriefed, and received payment through Prolific.

### Materials

**Fig. 12:**
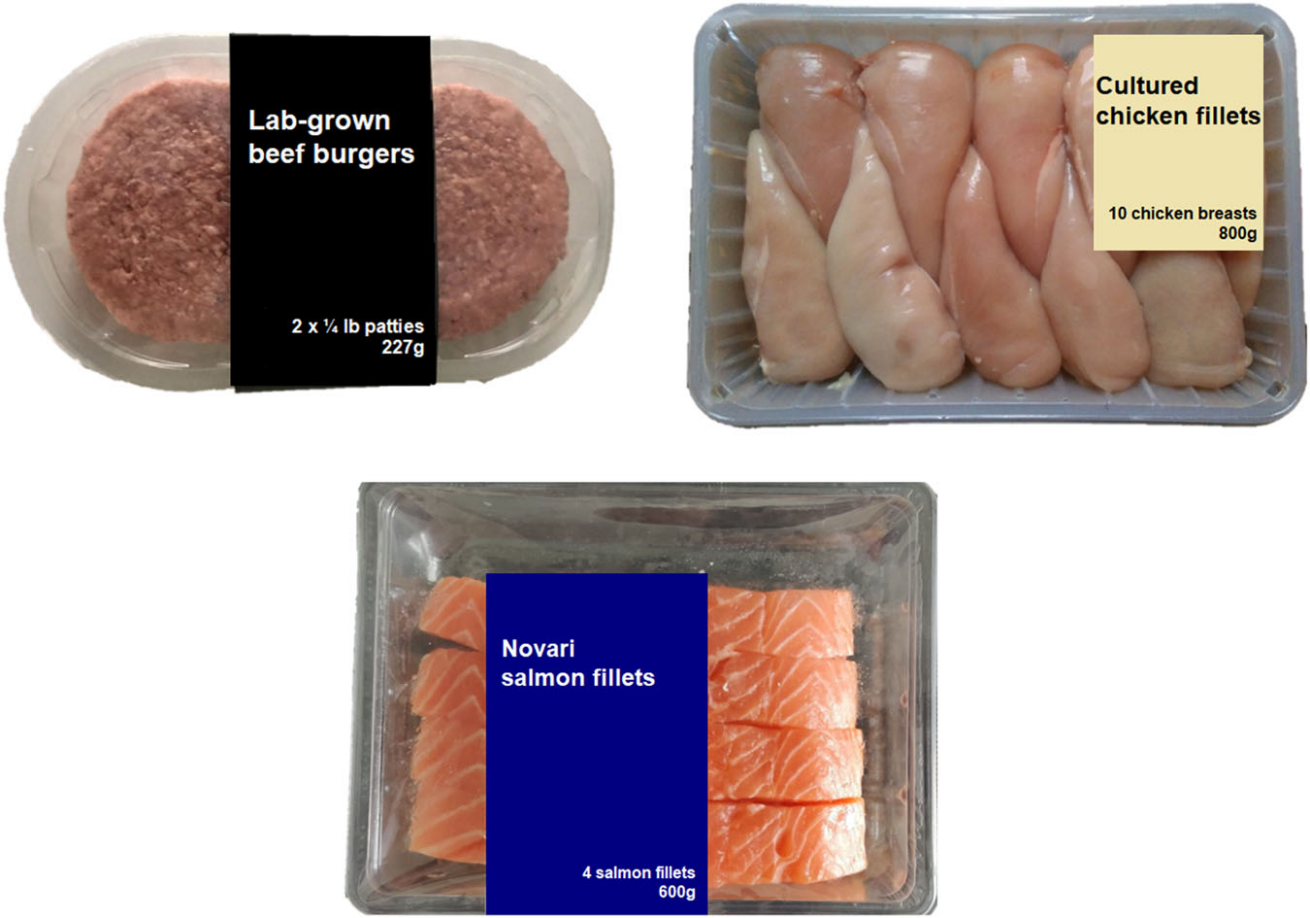
Samples of the images used for beef, chicken, and salmon with different labels. Image created by Christopher Bryant utilizing images from world.openfacts.org (Creative Commons licenses, via Google Images). These images were shown with varied labels according to experimental condition.

Images used were adapted from those found via copyright-free searches on Google Images. The original images had Creative Commons licences on the website world.openfacts.org (see Fig. 12).

As well as testing a variety of labels for these products, we also included a descriptive term, which succinctly describes the product — ‘Grown from [animal] cells, not farmed [or fished]’. This term was included as a benchmark for the other names in terms of product understanding and appeal, and provided an additional level of comparison to the research conducted by Hallman and Hallman^30,31^.

The full survey instrument is included in the Appendix A.

### Analyses

In order to compare the impact of different labels on consumer perceptions of cell-cultivated beef, chicken, and salmon, we employed a series of ANOVA and chi square analyses. Analyses were conducted using SPSS Version 26. All differences were considered statistically significant where *p* < 0.05.

Appeal, purchase likelihood, and safety were rated on 1–5 scales, and were compared between naming conditions using a series of one-way ANOVAs within each product category. Pairwise differences were assessed using Tukey’s HSD. Product identification and perceived safety for allergy-sufferers were both assessed using categorical measures and were compared between naming conditions using chi square analyses within each product category.

### Reporting summary

Further information on research design is available in the Nature Portfolio Reporting Summary linked to this article.

## Supplementary information

Supplementary Material

Reporting summary

## Data Availability

The data that support the findings of this study are available from the author, C.B., upon request.
