# Author Correction: Nomenclature of cell-cultivated meat & seafood products

**DOI:** 10.1038/s41538-023-00177-3

**Published:** 2023-01-13

**Authors:** Marlana Malerich, Christopher Bryant

**Affiliations:** 1 School of Geosciences, University of Edinburgh, Edinburgh, UK; 2 Department of Psychology, University of Bath, Bath, UK

**Keywords:** Technology, Science, technology and society

Correction to: *npj Science of Food* 10.1038/s41538-022-00172-0, published online 10 December 2022

In the original article Table 4 was inadvertently presented as a duplicate of Table 5, and the correct version of Table 4 was missing from the manuscript. The article has now been corrected and the duplicate Table 5 has been removed, and the correct Table 4 is now included.

