# Supplementary material for: Nomenclature of cell-cultivated meat & seafood products
Source: NPJ Sci Food. 2022 Dec 10;6:56. doi: 10.1038/s41538-022-00172-0 (PMC9734853; doi:10.1038/s41538-022-00172-0)
Supplement: Supplementary file 1 — Supplementary Material [file 41538_2022_172_MOESM1_ESM.pdf]

Appendices

Appendix A: Full survey instrument

1. Information & Consent Page

Information for participants

Background

We invite you to take part in this study, which aims to understand how people understand a new type of food product. Your participation will help us understand the best ways to label and discuss these products.

Study procedure

The questionnaire will take just 5 minutes to complete. First, you will be asked two verification questions, which are designed to remove automated or low-quality responses. They are not trick questions - please answer them as instructed. Next, you will see an image of the food product, and answer some questions about the product. Finally, you will answer some basic demographic questions.

Data handling

All the data you give through this survey will be anonymised. This means that any identifying information will be removed and you will not be able to be identified in the documenting or reporting of this study. However, this also means that you will not be able to withdraw your answers after finishing the questionnaire.

This questionnaire is distributed through Qualtrics. Qualtrics encrypts your data using Transport Layer Security (TLS). In accordance with the UK's Data Protection Act, this data is then managed by the University of Bath and will be made 'Open Access' (see below) when the study is completed.

What is Open Access?

Open Access data is made available to people who may want to analyse the data or are interested in the research, at no cost. This means we will not be able to control how this data is used, however, your data will be anonymised (as explained above).

Why Open Access?

By making data Open Access, the impact of investment is increased as the data can be used more widely and it encourages more, different, research. As much research is publicly funded, the research outcomes should be available to the public. Therefore, Open Access is often required by scientific journals and funders and is considered to be best practice.

Other information

The University of Bath Department of Psychology Ethics Committee has approved this study. Completing this questionnaire will not involve any risks beyond those normally experienced in your daily life. Involvement in this research is voluntary. You can, at any point, withdraw from the study. You do not need to give an explanation and will not experience any negative effects as a result of withdrawing.

At the end of the questionnaire you will be given more information about the study.

If you have any concerns about being involved in this study, please contact:

Christopher Bryant, Principal Investigator
Department of Psychology, University of Bath
C.J.Bryant@bath.ac.uk

1. Please check the box to continue to the study.

    1. I have read and understood this page, and I agree to take part in the study.

2. Attention Check

2. Qualtrics ReCAPTCHA

3. Product opinions

Imagine you are at your local supermarket shopping for some **fish** and you come across the following item:

> ***Image will differ between 27 possible product combinations:***
>
> ***9 LABELS: [Cell-cultivated; Cultivated; Cell-cultured; Cultured; Cell-based; Lab-grown; Artificial; Novari; 'Grown from cells, not farmed or fished']***
>
> ***3 PRODUCTS: [Salmon fillet; Chicken fillet; Beef burger]***

3. Based on the information shown above, what does it communicate about this tuna?

   a. **It is a fillet of a fish that has been caught wild**
   b. **It is a fillet of a fish that has been farm raised**
   c. **It is a fillet of a fish produced by growing fish cells in a food facility**
   d. **It is a plant-based fillet of fish**
   e. **Don't know/Unsure**

4. To what extent does this product sound appealing?
   a. Not at all appealing
   b. Somewhat unappealing
   c. Neither appealing not unappealing
   d. Somewhat appealing
   e. Very appealing

5. How likely would you be to purchase this product?
   a. Extremely unlikely
   b. Somewhat unlikely
   c. Neither likely nor unlikely
   d. Somewhat likely
   e. Extremely likely

6. **Are you a robot and/or paying attention?**
   a. **I am a robot**
   b. **I am a human and I am paying attention**
   c. **I am a human but I am not paying attention**
   d. **I am a robot and I am not paying attention**

7. If one were ***not allergic*** to **fish**, how safe do you think it is to eat this **tuna**?
   a. Very unsafe
   b. Somewhat unsafe
   c. Neither safe nor unsafe
   d. Somewhat safe
   e. Very safe
   f. Don't know/Unsure

8. Would it be safe to eat this **tuna** if one were ***allergic*** to **fish**?
   a. Yes
   b. No
   c. Don't know/Unsure

4. Demographics

9. How do you identify yourself?
    a. Male
    b. Female
    c. Other
    d. Prefer not to answer

10. How old are you?

    a. Under 18
    b. 18 - 24
    c. 25 - 34
    d. 35 - 44
    e. 45 - 54
    f. 55 - 64
    g. 65+
    h. Prefer not to answer

11. For quality control purposes, please select "Yellow" from the list below.
    a. Blue
    b. Yellow
    c. Red
    d. Green

12. Which of the following best described your total annual household income before taxes?
    a. Less than $25,000
    b. $25,000 - $49,999
    c. $50,000 - $74,999
    d. $75,000 - $99,999
    e. $100,000 - $149,999
    f. $150,000 - $199,999
    g. $200,000 or more
    h. Prefer not to answer

13. Which region of the U.S do you live in?
    a. Northeast (CT, ME, MA, NH, RI, VT, NJ, NY, PA)
    b. South (AL, AR, FL, GA, KY, LA, MI, NC, SC, TN, VA, WV, MD, DE, OK, TX, DC)
    c. Mid-West (IL, IN, IA, KS, MI, MN, MO, NE, ND, OH, SD, WI)
    d. West (AK, CO, CA, HI, ID, MT, NV, OR, UT, WA, WY, NM, AZ)

5. Debriefing

<u>Thank you for taking part!</u>

This study is designed to investigate how people perceive different labels for cell-cultivated meat and seafood products. In particular, we investigated the level of understanding, perceived allergenicity, and consumer appeal associated with a range of different labels for these products.

We greatly appreciate your involvement in this study. If you have any questions or concerns, or would like to find out about the results of this research, please contact the researcher.

Thank you.

Researcher:

Christopher Bryant
Department of Psychology, University of Bath
C.J.Bryant@bath.ac.uk

Research Ethics Officer

Department of Psychology, University of Bath
psychology-ethics@bath.ac.uk

PRODUCT IMAGES - From world.openfacts.org (Creative Commons licenses, via Google Images)

<table>
<tr><th></th><th>Salmon fillet 3</th><th>Chicken fillet 2</th><th>Beef burger 1</th></tr>
<tr><td>Cell-cultivated 1</td><td>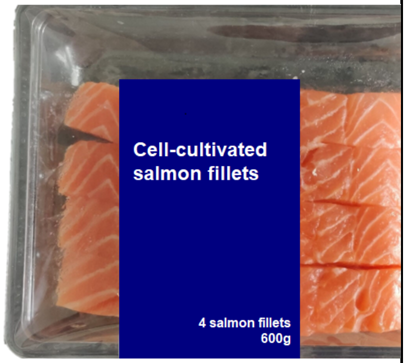
</td><td>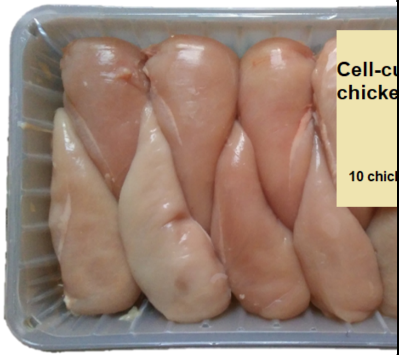
</td><td>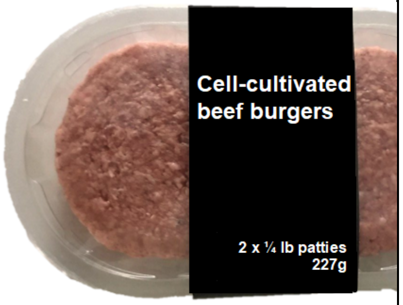
</td></tr>
<tr><td>Cultivated 2</td><td>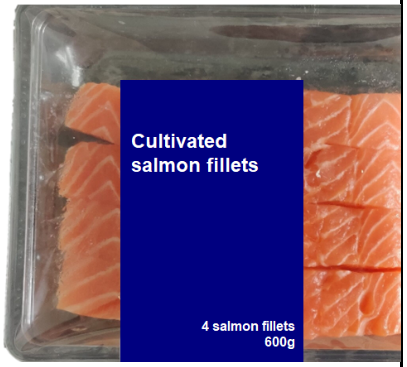
</td><td>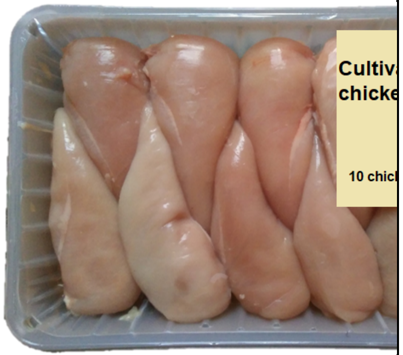
</td><td>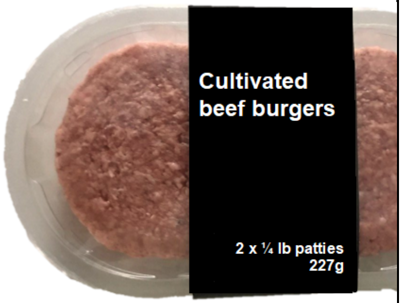
</td></tr>
</table>

<table>
<tr><td>Cell-cultur
ed
3</td>
<td>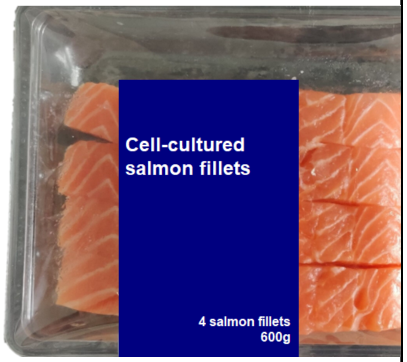
</td>
<td>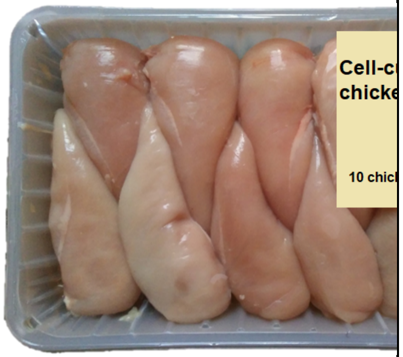</td>
<td>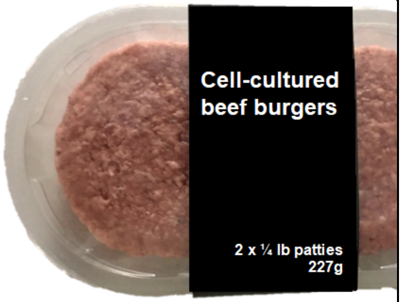
</td></tr>
<tr><td>Cultured
4</td>
<td>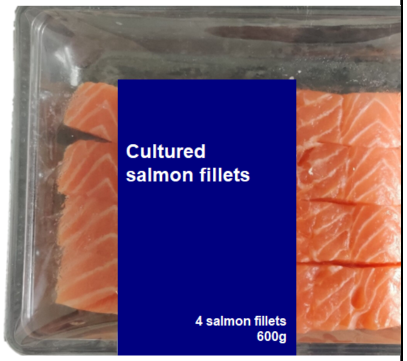
</td>
<td>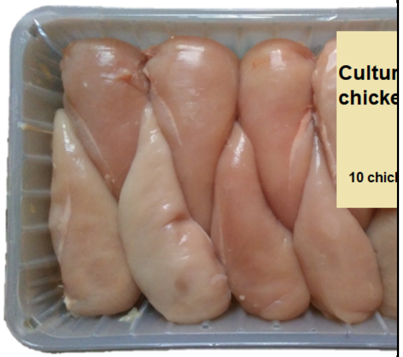</td>
<td>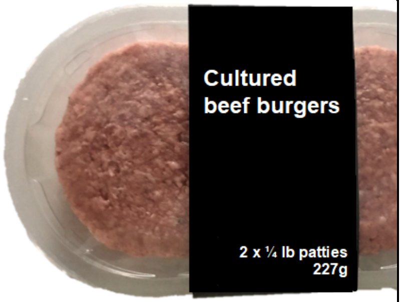
</td></tr>
<tr><td>Cell-based
5</td>
<td>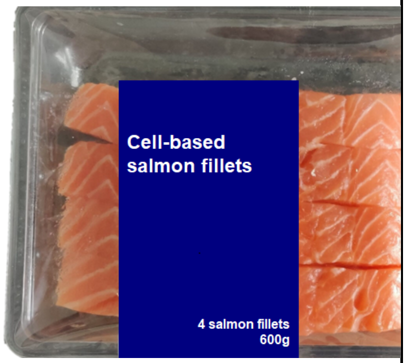
</td>
<td>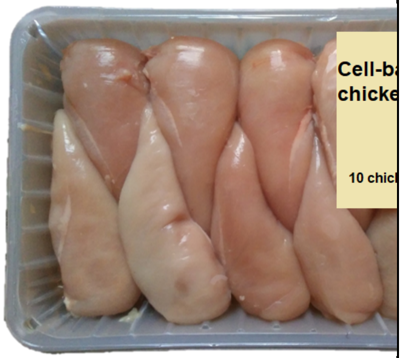</td>
<td>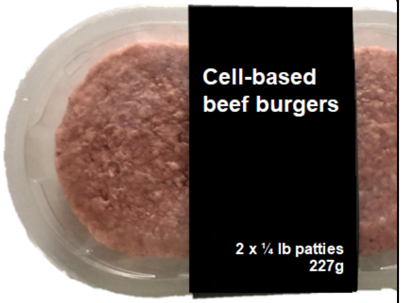
</td></tr>
</table>

**Novari**<br>**6**	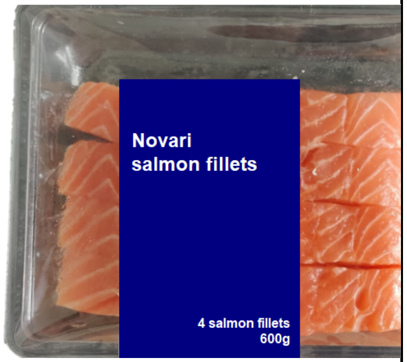	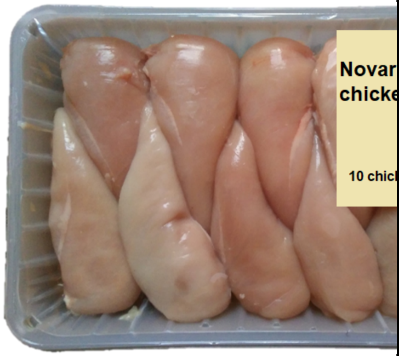	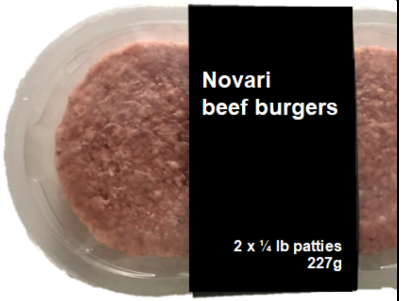
**Lab-grown**<br>**7**	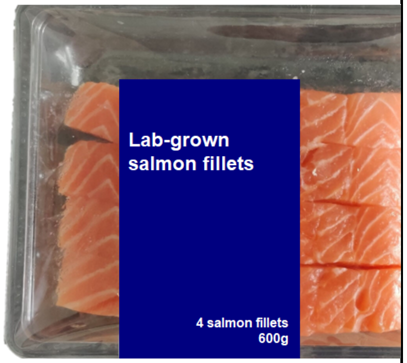	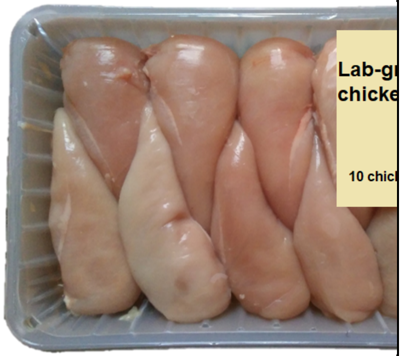	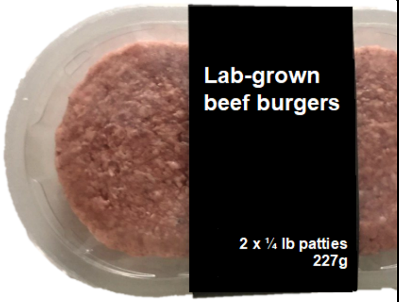
**Artificial**<br>**8**	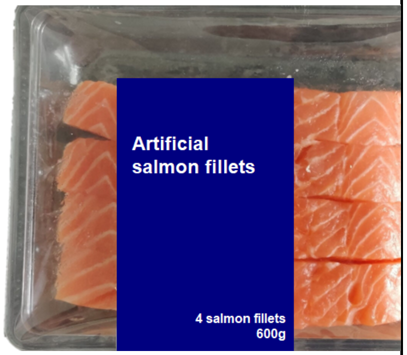	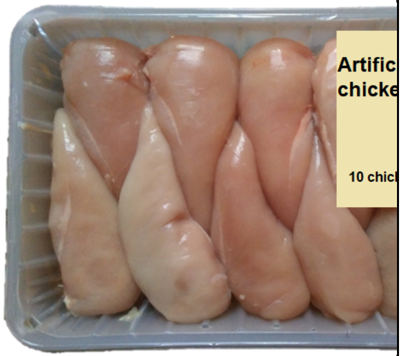	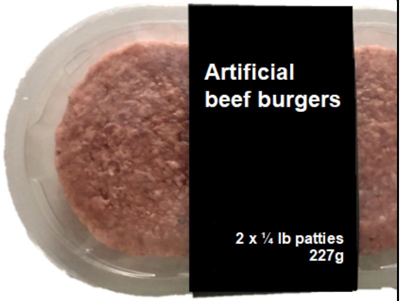

**Control 9**	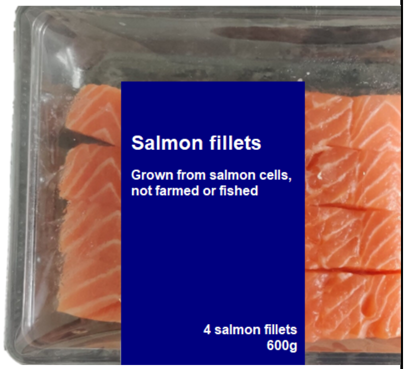Salmon fillets<br>Grown from salmon cells, not farmed or fished<br>4 salmon fillets 600g	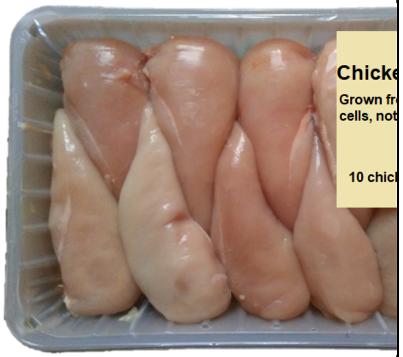Chicke<br>Grown fr<br>cells, not<br>10 chic	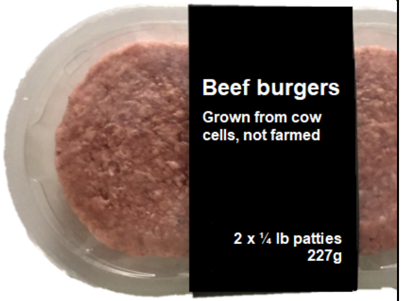Beef burgers<br>Grown from cow cells, not farmed<br>2 x ¼ lb patties 227g
