# Supplementary material for: Nomenclature of cell-cultivated meat & seafood products
Source: NPJ Sci Food. 2022 Dec 10;6:56. doi: 10.1038/s41538-022-00172-0 (PMC9734853; doi:10.1038/s41538-022-00172-0)
Supplement: Supplementary file 2 — Reporting summary [file 41538_2022_172_MOESM2_ESM.pdf]

nature portfolio

Corresponding author(s): Marlana Malerich

Last updated by author(s): Oct 12, 2022

Reporting Summary

Nature Portfolio wishes to improve the reproducibility of the work that we publish. This form provides structure for consistency and transparency in reporting. For further information on Nature Portfolio policies, see our Editorial Policies and the Editorial Policy Checklist.

Statistics

For all statistical analyses, confirm that the following items are present in the figure legend, table legend, main text, or Methods section.

n/a	Confirmed	
☐	☒	The exact sample size (n) for each experimental group/condition, given as a discrete number and unit of measurement
☐	☒	A statement on whether measurements were taken from distinct samples or whether the same sample was measured repeatedly
☐	☒	The statistical test(s) used AND whether they are one- or two-sided<br>*Only common tests should be described solely by name; describe more complex techniques in the Methods section.*
☐	☒	A description of all covariates tested
☐	☒	A description of any assumptions or corrections, such as tests of normality and adjustment for multiple comparisons
☐	☒	A full description of the statistical parameters including central tendency (e.g. means) or other basic estimates (e.g. regression coefficient) AND variation (e.g. standard deviation) or associated estimates of uncertainty (e.g. confidence intervals)
☐	☒	For null hypothesis testing, the test statistic (e.g. F, t, r) with confidence intervals, effect sizes, degrees of freedom and P value noted<br>*Give P values as exact values whenever suitable.*
☒	☐	For Bayesian analysis, information on the choice of priors and Markov chain Monte Carlo settings
☒	☐	For hierarchical and complex designs, identification of the appropriate level for tests and full reporting of outcomes
☐	☒	Estimates of effect sizes (e.g. Cohen's d, Pearson's r), indicating how they were calculated

Our web collection on statistics for biologists contains articles on many of the points above.

Software and code

Policy information about availability of computer code

Data collection	Data was collected via Qualtrics from respondents recruited via Prolific.
Data analysis	Data was analysed using SPSS V28.

For manuscripts utilizing custom algorithms or software that are central to the research but not yet described in published literature, software must be made available to editors and reviewers. We strongly encourage code deposition in a community repository (e.g. GitHub). See the Nature Portfolio guidelines for submitting code & software for further information.

Data

Policy information about availability of data

All manuscripts must include a data availability statement. This statement should provide the following information, where applicable:

- Accession codes, unique identifiers, or web links for publicly available datasets
- A description of any restrictions on data availability
- For clinical datasets or third party data, please ensure that the statement adheres to our policy

Data and syntax are available on OSF here: https://osf.io/v6tyg/

Human research participants

Policy information about studies involving human research participants and Sex and Gender in Research.

Reporting on sex and gender	We refer to gender consistently in the manuscript. We report the percentage of participants who self-reported being male, female, or other gender across age groups. We also report how gender affected acceptance in a regression.
Population characteristics	We report the proportion of participants in each age group in Section 2.1: Participants.<br>Males 18-29: 10.8%; Females 18-29: 9.7%; Other 18-29: 0.5%<br>Males 30-44: 12.4%; Females 30-44: 12.8%; Other 30-44: 0.2%<br>Males 45-59: 12.1%; Females 45-59: 12:1%; Other 45-59: 0.1%<br>Males 60+: 13.2%; Females 60+: 15.9%; Other 60+: 0%
Recruitment	Participants were recruited via the survey platform Prolific, and were paid $1 for their participation in the study. Further details are in Section 2.1 and 2.2.
Ethics oversight	This study received ethical approval from the University of Bath Psychology Research Ethics Committee (PREC 22-100).

Note that full information on the approval of the study protocol must also be provided in the manuscript.

Field-specific reporting

Please select the one below that is the best fit for your research. If you are not sure, read the appropriate sections before making your selection.

☐ Life sciences ☒ Behavioural & social sciences ☐ Ecological, evolutionary & environmental sciences

For a reference copy of the document with all sections, see nature.com/documents/nr-reporting-summary-flat.pdf

Behavioural & social sciences study design

All studies must disclose on these points even when the disclosure is negative.

Study description	The study used a quantitative experimental cross-sectional survey design whereby participants were recruited to an online survey and randomly allocated to one of 27 (9x3) experimental conditions.
Research sample	Participants were users of the online survey platform, Prolific. The overall sample was representative of the US population in terms of age and gender. The age and gender breakdown was as follows:<br>Males 18-29: 10.8%; Females 18-29: 9.7%; Other 18-29: 0.5%<br>Males 30-44: 12.4%; Females 30-44: 12.8%; Other 30-44: 0.2%<br>Males 45-59: 12.1%; Females 45-59: 12:1%; Other 45-59: 0.1%<br>Males 60+: 13.2%; Females 60+: 15.9%; Other 60+: 0%
Sampling strategy	We used a stratified sampling method to ensure a representative proportion of participants from each gender/age group. This was achieved by obtaining the relevant proportions of the overall US population, and applying these proportions to our target sample size (2,700).
Data collection	Data was collected via the online survey platform Qualtrics. Participants filled out the survey on their own remotely.
Timing	Data was collected between July 27 2022 - August 07 2022.
Data exclusions	55 participants were removed for various reasons, including unfinished responses, speeding, not giving consent, failing attention check questions, and being under the age of 18.
Non-participation	21 participants did not complete the survey, and incomplete responses were removed. Participants were not able to take the survey if they did not indicate their consent to take part.
Randomization	Participants were allocated at random to (a) one of 3 types of meat, and (b) one of 9 possible labels. Both randomization procedures were carried out using Qualtrics' built-in randomisation features.

Reporting for specific materials, systems and methods

We require information from authors about some types of materials, experimental systems and methods used in many studies. Here, indicate whether each material, system or method listed is relevant to your study. If you are not sure if a list item applies to your research, read the appropriate section before selecting a response.

Materials & experimental systems

n/a	Involved in the study
☒	☐ Antibodies
☒	☐ Eukaryotic cell lines
☒	☐ Palaeontology and archaeology
☒	☐ Animals and other organisms
☒	☐ Clinical data
☒	☐ Dual use research of concern

Methods

n/a	Involved in the study
☒	☐ ChIP-seq
☒	☐ Flow cytometry
☒	☐ MRI-based neuroimaging
